# Supplementary material for: Epithelial–Mesenchymal Transition Expression Profile Stratifies Human Glioma into Two Distinct Tumor-Immune Subtypes
Source: Brain Sci. 2023 Mar 5;13(3):447. doi: 10.3390/brainsci13030447 (PMC10046881; doi:10.3390/brainsci13030447)
Supplement: Supplementary file 1 [file brainsci-13-00447-s001.zip › Supplementary Figures and Tables.pdf]

Supplementary Figures

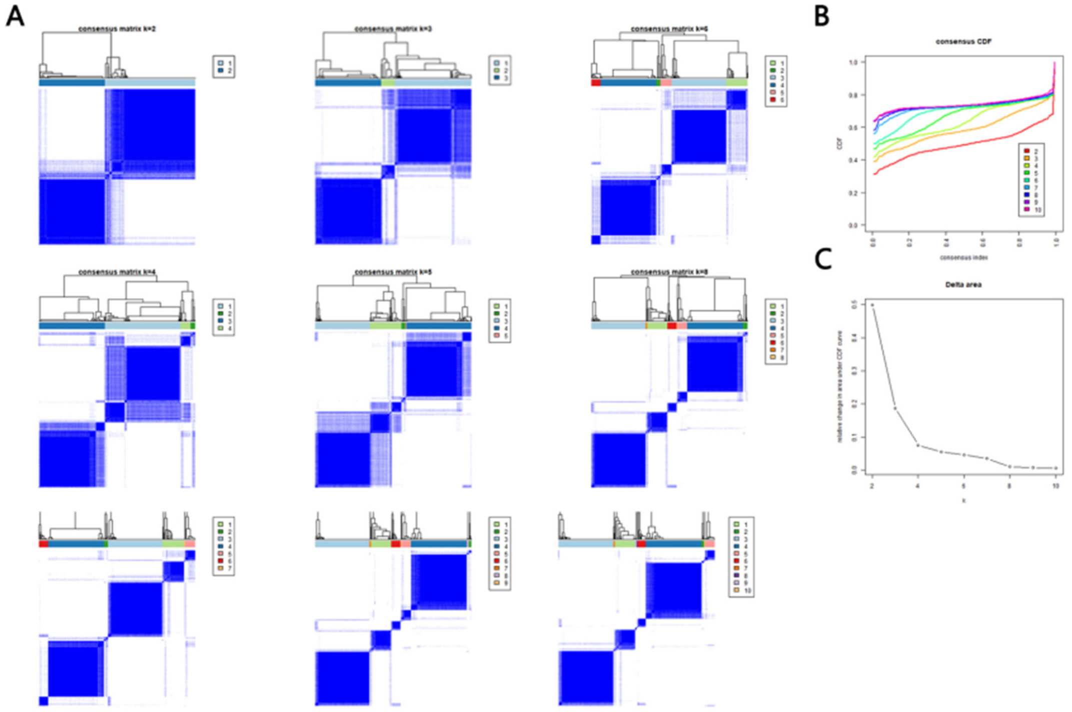

Figure S1. Consensus clustering based in metabolism gene expression of CGGA cohort1.

(A) Clustering matrix for k=2 to k=10. (B) CDF (cumulative distribution function) curve for k=2 to k=10. (C) Relative change in area under CDF curve for k=2 to k=10.

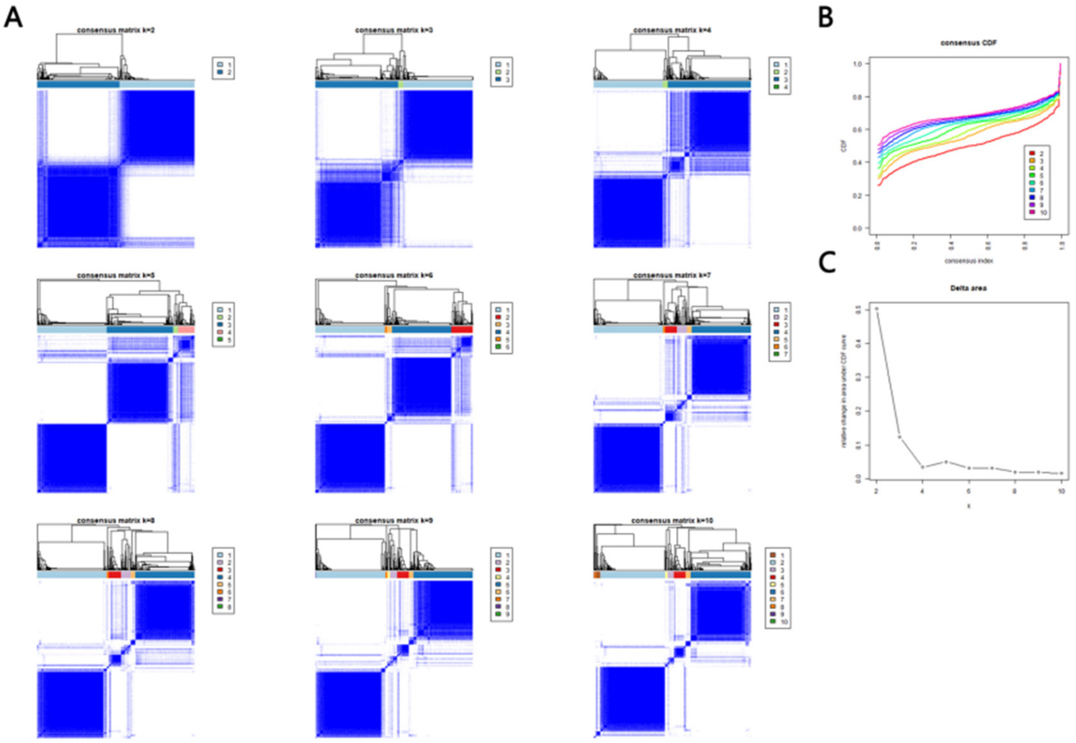

Figure S2. Consensus clustering based in metabolism gene expression of CGGA cohort2.

(A) Clustering matrix for k=2 to k=10. (B) CDF (cumulative distribution function) curve for k=2 to k=10. (C) Relative change in area under CDF curve for k=2 to k=10.

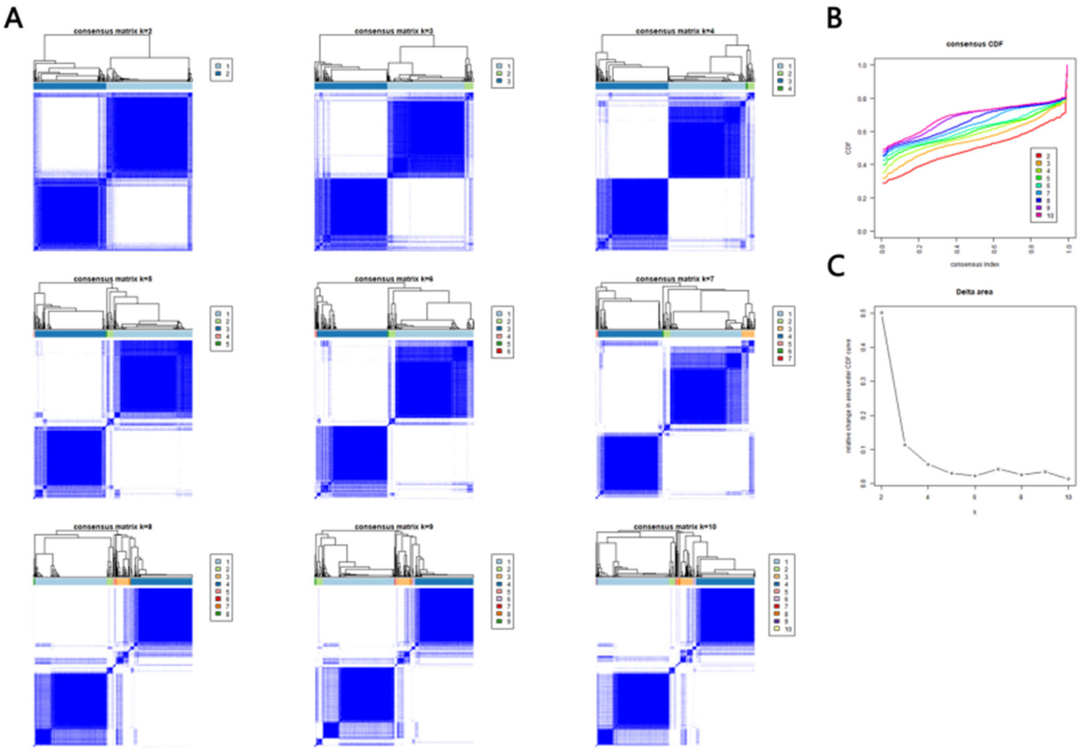

Figure S3. Consensus clustering based in metabolism gene expression of GLASS cohort.

(A) Clustering matrix for k=2 to k=10. (B) CDF (cumulative distribution function) curve for k=2 to k=10. (C) Relative change in area under CDF curve for k=2 to k=10.

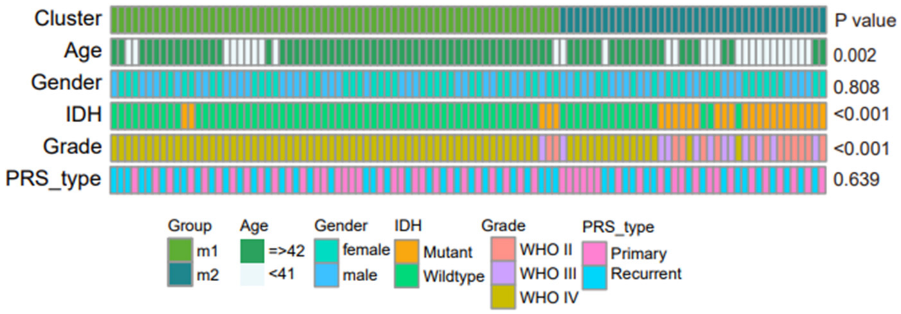

Figure S4. Clinical characteristics of EMT subtypes in GLASS cohort.

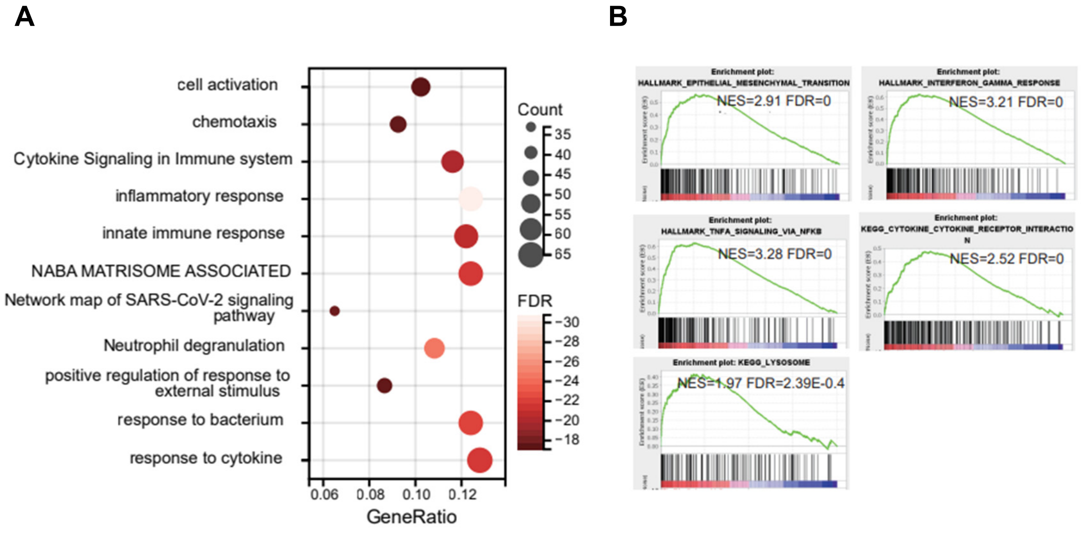

Figure S5. Functional enrichment analysis of the EMT subtypes in GLASS cohort.

(A) GO analysis of upregulated genes in m1 subtype. (B) Enriched functions of m1 subtype identified through GSEA.

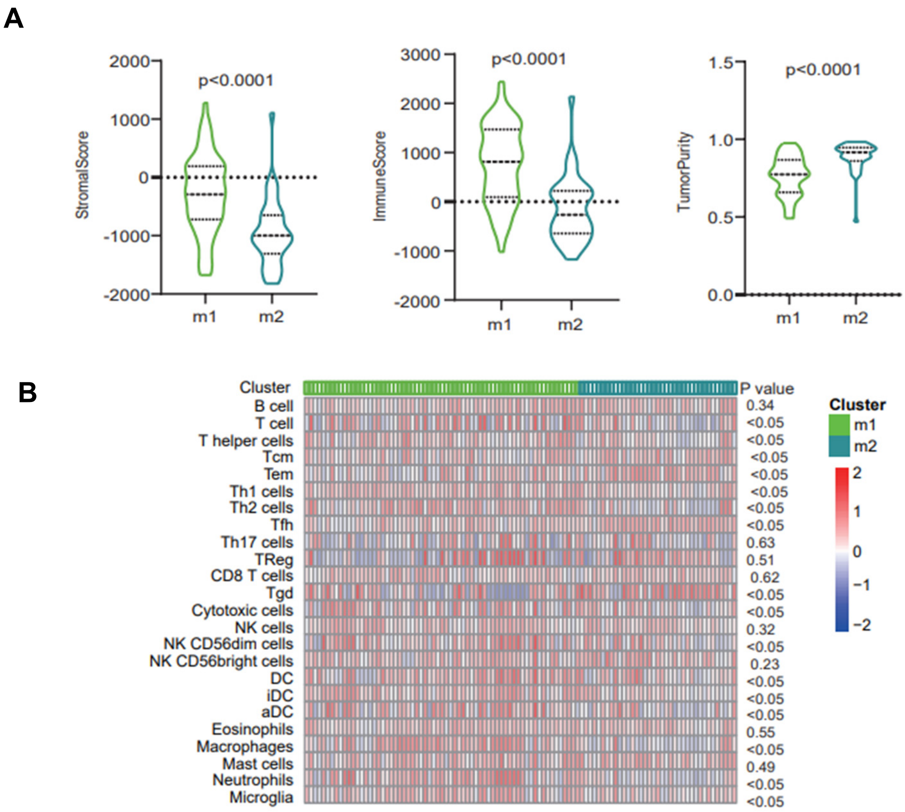

Figure S6 Immune infiltration of two subtypes in the GLASS cohort.

(A)Violin plots of immune, stromal and tumor purity scores from ESTMATE (ANOVA test). B Heatmaps show differential enrichments of immune-related signatures in two subtypes. ANOVA test was used for statistical analysis, and the P values were labeled.

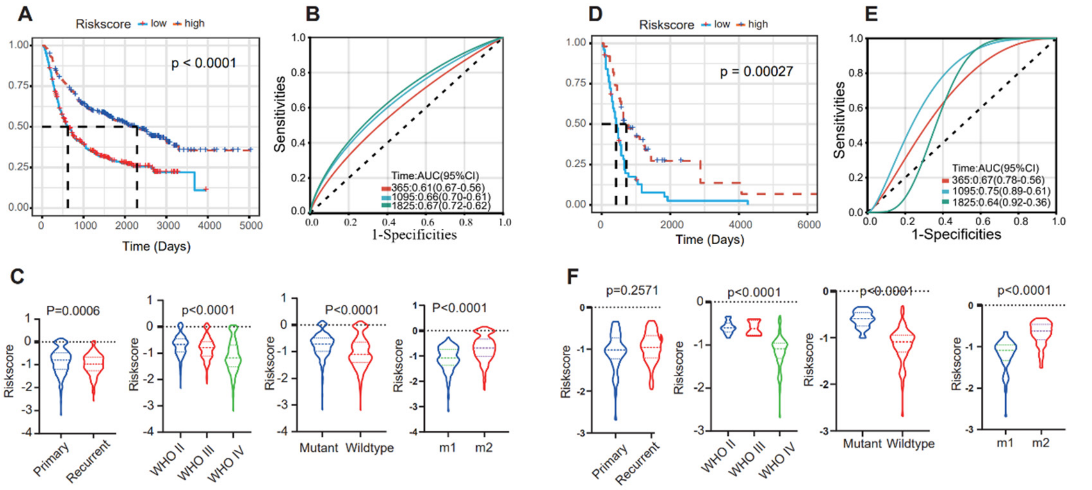

Figure S7 Identification and analysis of the EMT related signature.

(A) Distribution of the risk score, overall survival (OS) and expression level of 11 genes in the risk signature. (B)Kaplan–Meier survival analysis of the EMT signature inpatients of CGGA cohort1.E The time receiver operating characteristic (ROC) curve analyses to predict 1-, 3-, and 5-y OS according to risk score in CGGA cohort1 dataset. (C)Distribution of the risk score in glioma patients stratified by WHO grade.

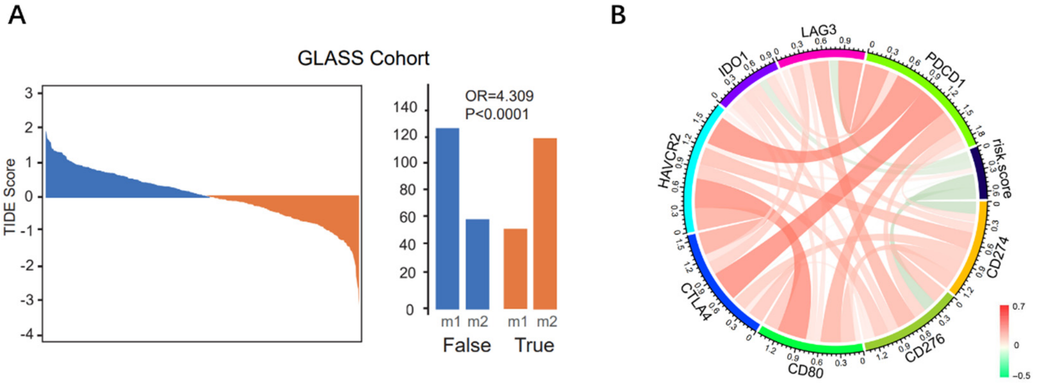

Figure S8 The risk signature is associated with ICB response and immune checkpoint in GLASS Cohort. (A) The TIDE score and response results to immunotherapy of patients with glioma. (B) The correlation coefficient between risk score and immune checkpoints

Supplementary Tables

Table S1. Clinical characteristics of patients in this study.

**Variable**	**CGGA cohort1**<br>n=325	**CGGA cohort2**<br>n=693	**GLASS cohort**<br>n=102
**Age**			
≥ 42 years	175	370	24
< 42 years	150	323	78
NA	0	1	0
**Gender**			
Female	122	295	54
Male	203	398	48
**IDH**			
Mutant	175	356	26
WT	149	286	76
NA	1	51	0
**1P/19Q**			
Codeleted	67	145	6
Non-codeleted	250	478	98
NA	8	70	0
**MGMT promoter**			
Methylated	157	315	49
Unmethylated	149	151	28
NA	19	227	25
**Grade**			
II	103	188	14
III	79	255	12
IV	139	249	76
NA	4	1	0
